# Reply to Comments on the "Mortality Rate and Years of Life Lost Due to Burns in Southern Iran During 2004–2019: A Population-Based Study"

**DOI:** 10.34172/aim.2024.09

**Published:** 2024-01-01

**Authors:** Habibollah Azarbakhsh, Leila Moftakhar, Seyed Sina Dehghani, Jafar Hassanzadeh, Seyed Parsa Dehghani, Alireza Mirahmadizadeh

**Affiliations:** ^1^Student Research Committee, Shiraz University of Medical Sciences, Shiraz, Iran; ^2^Student Research Committee, Shiraz University of Medical Sciences, Shiraz, Iran; ^3^School of Medicine, Shiraz University of Medical Sciences, Shiraz, Iran; ^4^Research Center for Health Sciences, Institute of Health, Department of Epidemiology, Shiraz University of Medical Sciences, Shiraz, Iran; ^5^School of Medicine, Shiraz University of Medical Sciences, Shiraz, Iran; ^6^Non-Communicable Diseases Research Center, Shiraz University of Medical Sciences, Shiraz, Iran

## Reply,

 We thank Rostami et al^1^ for their valuable advice in the recent report published in *Archives of Iranian Medicine*.

 Considering that the population of the age group of 15–29 years is more than the population under the age of 5, one of the possible reasons is the high number of deaths in this age group.

 There are two primary sources to obtain mortality information in Iran, including the Electronic Death Registration System (EDRS) from the Ministry of Health and Medical Education (MOHME) and, at the national level, the National Organization for Civil Registration (NOFCR). In Iran, first, trained physicians report deaths and then codify the causes of death according to the national protocol and the International Classification of Diseases (ICD). Afterward, hospitals, local health centers, cemeteries, and forensic organizations report these data monthly to the Death Registration Committee, reducing the possibility of undercounting and errors.

 Burn deaths and self-immolation are coded differently, although self-immolation may be underestimated. However, the necessary checks have been performed, and this possibility has been minimized.
